# Emerging Severe Acute Respiratory Syndrome Coronavirus 2 Mutation Hotspots Associated With Clinical Outcomes and Transmission

**DOI:** 10.3389/fmicb.2021.753823

**Published:** 2021-10-18

**Authors:** Xianwu Pang, Pu Li, Lifeng Zhang, Lusheng Que, Min Dong, Bo Xie, Qihui Wang, Yinfeng Wei, Xing Xie, Lanxiang Li, Chunyue Yin, Liuchun Wei, Kexin Huang, Yiming Hua, Qingniao Zhou, Yingfang Li, Lei Yu, Weidong Li, Zengnan Mo, Maosheng Zhang, Jing Leng, Yanling Hu

**Affiliations:** ^1^Guangxi Collaborative Innovation Center for Biomedicine, Guangxi Medical University, Nanning, China; ^2^PFOMIC Bioinformatics Company, Nanning, China; ^3^Guangxi Key Laboratory of Translational Medicine for Treating High-Incidence Infectious Diseases With Integrative Medicine, Guangxi University of Chinese Medicine, Nanning, China; ^4^Department of Virology II, National Institute of Infectious Diseases, Tokyo, Japan; ^5^School of Pharmacy, Guangxi Medical University, Nanning, China; ^6^School of Information and Management, Guangxi Medical University, Nanning, China; ^7^School of Basic Medical Sciences, Guangxi Medical University, Nanning, China; ^8^Life Sciences Institute, Guangxi Medical University, Nanning, China; ^9^The First Affiliated Hospital of Guangxi Medical University, Guangxi Medical University, Nanning, China; ^10^Center for Genomic and Personalized Medicine, Guangxi Medical University, Nanning, China

**Keywords:** SARS-CoV-2, COVID-19, mutation hotspots, asymptomatic, symptomatic

## Abstract

Severe acute respiratory syndrome coronavirus 2 (SARS-CoV-2) is the cause of the ongoing coronavirus disease 2019 (COVID-19) pandemic. Understanding the influence of mutations in the SARS-CoV-2 gene on clinical outcomes is critical for treatment and prevention. Here, we analyzed all high-coverage complete SARS-CoV-2 sequences from GISAID database from January 1, 2020, to January 1, 2021, to mine the mutation hotspots associated with clinical outcome and developed a model to predict the clinical outcome in different epidemic strains. Exploring the cause of mutation based on RNA-dependent RNA polymerase (RdRp) and RNA-editing enzyme, mutation was more likely to occur in severe and mild cases than in asymptomatic cases, especially A > G, C > T, and G > A mutations. The mutations associated with asymptomatic outcome were mainly in open reading frame 1ab (ORF1ab) and N genes; especially R6997P and V30L mutations occurred together and were correlated with asymptomatic outcome with high prevalence. D614G, Q57H, and S194L mutations were correlated with mild and severe outcome with high prevalence. Interestingly, the single-nucleotide variant (SNV) frequency was higher with high percentage of nt14408 mutation in RdRp in severe cases. The expression of ADAR and APOBEC was associated with clinical outcome. The model has shown that the asymptomatic percentage has increased over time, while there is high symptomatic percentage in Alpha, Beta, and Gamma. These findings suggest that mutation in the SARS-CoV-2 genome may have a direct association with clinical outcomes and pandemic. Our result and model are helpful to predict the prevalence of epidemic strains and to further study the mechanism of mutation causing severe disease.

## Introduction

Coronavirus disease 2019 (COVID-19) was caused by the severe acute respiratory syndrome coronavirus 2 (SARS-CoV-2). COVID-19 has rapidly spread worldwide and became a global health emergency. The clinical spectrum of SARS-CoV-2 infection appears to be wide, encompassing asymptomatic infection, mild upper respiratory tract illness, and severe viral pneumonia that may result in respiratory failure and even death, with many patients being hospitalized with pneumonia (Chen et al., 2020; Huang et al., 2020; Wang et al., 2020). According to the World Health Organization, the case fatality rate as of October 2020 was about 2.7%. However, the case fatality rate was up to 26% in severe cases (Grasselli et al., 2020). The ongoing rapid spread of the virus worldwide, coupled with asymptomatic cases and patients with severe symptoms, raises an important concern that it may further mutate into more highly transmissible or virulent forms.

Case fatality rates can widely vary according to geography, demographics, and healthcare infrastructure (Team, 2020), as well as according to various host factors, including advanced age, being male, and comorbidities (Docherty et al., 2020; Gupta et al., 2020). However, the viral factors underlying the severity of COVID-19 and the corresponding case fatality rate remain unclear. Some mutations in the SARS-CoV-2 genome were reportedly remarkably changed the virus’s properties, such as transmission modes and rates, as well as the ability to cause diseases (Kaushal et al., 2020; Zhou et al., 2020; Francisco et al., 2021). The D614G variant in the S glycoprotein was associated with increased transmissibility, infectivity, and viral loads but not with disease severity (Korber et al., 2020; Zhou et al., 2020). Parinita et al. (Majumdar and Niyogi, 2020) suggested that the presence of P25L in ORF3a was a probable mechanism of immune evasion and likely contributes to enhanced virulence, which was associated with higher case fatality rates in SARS-CoV-2 infection. Similarly, some mutations, such as S197L, S194L, and a 382-nucleotide deletion, were associated with clinical outcomes (Young et al., 2020; Nagy et al., 2021). Additionally, mutation types, such as the G/T variant in the open reading frame 1ab (ORF1ab) gene, was associated with clinical infections (Yildirim et al., 2021).

Therefore, RNA virus mutations not only contributed to viral adaptation that created a balance between the integrity of genetic information and genome variability (Domingo and Holland, 1997; Domingo, 2000; Lauring and Andino, 2010) but also led to different clinical outcomes. Understanding the characteristics of viral mutations is essential for the pathogenesis, immune escape, drug resistance, vaccine design, antiviral therapy, and diagnosis of COVID-19. In this context, we focused on SARS-CoV-2 mutations associated with clinical outcomes. First, 209,551 high-coverage complete virus sequences from the GISAID database were compared with the SARS-CoV-2 reference genome (NC_045512.2). This study aimed to gain important insights into virus mutations and their occurrence over time, especially hotspot mutations associated with different clinical outcomes. Second, RNA-dependent RNA polymerase (RdRp) and RNA-editing enzyme analyzed from 321 RNA-seq samples were used to explore the SARS-CoV-2 mutations that were associated with different clinical outcomes.

## Materials and Methods

### Data Source

Publicly available SARS-CoV-2 complete genomes of different patients were collected from the GISAID virus repository^1^ from January 1, 2020, to January 1, 2021. Viral sequences with a complete genome (28,000–30,000 bp) were included in this study. RNA-seq datasets available from the PRJNA601736 (two samples, raw sequence reads), PRJNA603194 (one sample, raw sequence reads), PRJNA605907 (eight samples, raw sequence reads), PRJNA631753 (62 samples, raw sequence reads), PRJNA639791 (six samples, raw sequence reads), PRJNA656568 (93 SARS-CoV-2-positive samples, 99 samples without any virus infection, transcriptome, or gene expression), and PRJNA683226 (37 mild samples, 10 moderate samples, three severe samples, transcriptome, or gene expression) projects were also downloaded from the National Center for Biotechnology Information (NCBI)^2^.

### Single-Nucleotide Variation Calling in Genomic Sequence

Single-nucleotide variants (SNVs) were called with a custom R script, by comparing the viral genome sequences with the reference sequence. The reference of SARS-CoV-2 is NC_045512.2 sequence, that of SARS is NC_004718.3 sequence, and that of Middle East respiratory syndrome (MERS) is NC_019843.3 sequence. SNVs occurring on coding sequences were annotated with custom R scripts to determine the outcome of nucleotide changes, including nonsense, non-synonymous, and synonymous mutations.

### Single-Nucleotide Variant Calling in the Transcriptome of Severe Acute Respiratory Syndrome Coronavirus 2

REDItools 2 (Picardi and Pesole, 2013; Flati et al., 2020) and JACUSA (Piechotta et al., 2017) were used together to call the SNVs in the transcriptome of SARS-CoV-2. With REDItools 2, all SNVs within 15 nucleotides from the beginning or the end of the reads were removed to avoid artifacts due to misalignments. Furthermore, the AS_StrandOddsRatio parameter was utilized to avoid potential artifacts due to strand bias, and any mutation with an AS_StrandOddsRatio of >4 was removed from the dataset. The overlap of mutations generated by REDItools 2 and JACUSA was considered. The threshold to filter the SNVs was based on minimum coverage (20 reads), number of supporting reads (at least four mutated reads), allelic fraction (0.5%), quality of the mapped reads (>25), and base quality (>35).

### Gene Expression in Transcriptome

Gene expression in transcriptome was analyzed by introducing transcript per million (TPM) to estimate gene expression levels. Given that TPM considers the effects of sequencing depth, gene length, and sample on reads count, it was often used to estimate gene expression levels. In this study, all samples were processed through a SARS-CoV-2 reference-based assembly pipeline that involved removing non-SARS-CoV-2 reads with Kraken2 (Wood et al., 2019) and aligning to the SARS-CoV-2 reference genome NC_045512.2 by using Samtools (Li et al., 2009). SARS-CoV-2 TPM was calculated using the R package tximport (Soneson et al., 2015) via the length scaled TPM method.

### Model for Predicting Clinical Outcome

A total of 1,329 sequences with clinical information were divided into symptomatic (84.27%) and asymptomatic (15.73%) outcomes. These mutations with statistical significance between asymptomatic and symptomatic outcomes were input to the model after eliminating the influence of confounding variable. After trying these common dichotomous classifiers, including Nearest Neighbors, Gaussian Process, Decision Tree, Random Forest, AdaBoost, Naive Bayes, support vector machine (SVM) Linear, SVM radial basis function (RBF), and SVM Sigmoid, the neural network performed the best for the data. So we developed the model by using the neural network machine learning technology. The neural network consisted of input layer, hidden layer, and output layer. Each layer consisted of a fixed number of neurons, each of which could receive input and process or output. The output of each neuron was expressed by the following formula:


$${\text{h}}_{\text{W},\text{b}}(x)=f(W^{T}x)=f(\sum_{i=1}^{n}{\text{W}}_{\text{i}}x_{i}+b)$$

The ⨏ was called the activation function. In this article, the identity function was used as the activation function, and the formula was as follows:


f⁢(z)=x

In addition, mutations were sorted into matrices and cleaned and processed to obtain validation datasets, test datasets, and training datasets; and the three datasets were split in a ratio of 1:2:7. The model was trained with the training datasets; the accuracy and generalization ability of the model were assessed with test datasets; and the model parameters were tuned using validation datasets. At the same time, the training process of the model was monitored to prevent model overfitting. In the study, the hidden layer of the model had five layers, and each hidden layer had 256, 128, 64, 32, and 16 neurons. At last, these sequences without clinical information were predicted through the model.

### Statistical Analysis

The normality of data distribution was checked via the Shapiro–Wilk test. Categorical variables were expressed as absolute frequency and percentages. The different SNV counts per genome between different clinical outcomes was analyzed using one-way ANOVA followed by the Student–Newman–Keuls test, chi-square, and multivariate logistic regression were utilized to compare the frequency of mutation sites between different clinical outcomes. Wald test and log2 false discovery rate (FDR) were used to compare the number of gene expression between different groups. Pearson’s correlation coefficient was calculated to estimate the correlation between sample gene expression and viral load. A higher Pearson’s correlation coefficient indicated a stronger correlation. All *p*-values were calculated from two-sided tests using 0.05 as the significance level.

## Results

### Characterization of Mutations in the Severe Acute Respiratory Syndrome Coronavirus 2 Genome

**FIGURE 1:**
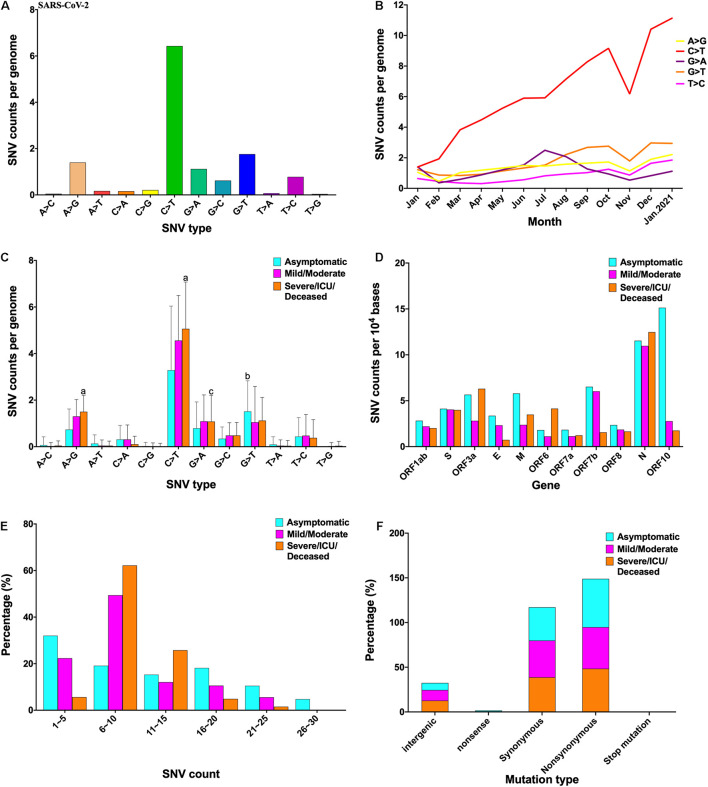
Characterization of mutations in the SARS-CoV-2 genome. **(A)** SNV type in the SARS-CoV-2 genome. **(B)** Dynamics of SNV counts over time. **(C)** Number of different SNV types distributed in the three clinical outcomes. **(D)** SNVs of three clinical outcomes distributed in the gene of SARS-CoV-2. **(E)** Ranges of SNV counts in the three clinical outcomes. **(F)** Mutation results of three clinical outcomes. ^a^*p* < 0.001, ^b^*p* < 0.01, and ^c^*p* < 0.05 represent that there is significant difference among the three clinical outcomes. All results were analyzed using one-way ANOVA followed by the Student–Newman–Keuls test. SARS-CoV-2, severe acute respiratory syndrome coronavirus 2; SNV, single-nucleotide variant.

A total of 209,551 complete SARS-CoV-2 genome sequences were downloaded from the GISAID database. All sequences were aligned and compared with the SARS-CoV-2 reference genome (NC_045512.2). Results showed that C > T, G > T, A > G, and G > A mutations were the major SNV types (Figure 1A), and the frequency of these SNV types increased, especially that of the C > T mutation from January 2020 to January 2021 (Figure 1B). The effects of clade, region, sex, and age on base mutations were also analyzed, indicating that clade and region were also related to mutation. For example, GV was more prone to C > T mutations, while GR was more prone to A > G, G > A, and G > T mutations. Furthermore, Europe and Africa were more prone to all base mutations than the other regions (Supplementary Figure 1).

A total of 1,329 high-quality sequences had special clinical information. According to clinical description, these sequences were divided into three groups: asymptomatic, mild/moderate, and severe/intensive care unit (ICU)/deceased. The mutation frequency of C > T (*p* < 0.001), A > G (*p* < 0.001), and G > A (*p* < 0.05) was higher in severe cases than in asymptomatic cases, whereas that of G > T (*p* < 0.01) was higher in asymptomatic cases than that in others (Figure 1C). S, ORF3a, M, ORF7b, and N genes were more likely to mutate in all cases; ORF10 was more likely to mutate in asymptomatic cases (Figure 1D). The count of SNV per genome was divided into six groups: asymptomatic cases were mainly distributed in 1–5 (32%) and 6–10 (19.1%), mild/moderate cases were largely distributed in 1–5 (22.3%) and 6–10 (49.4%), and severe cases were chiefly distributed in 6–10 (62.2%) and 11–15 (25.8%) (Figure 1E). The mutation results of asymptomatic, mild/moderate, and severe cases were primarily non-synonymous (54, 46.5, and 48.3%, respectively), followed by synonymous (37, 41.1, and 38.7%, respectively) (Figure 1F). The sequence context of mutation showed that the motif preference for the three clinical outcomes was different (Supplementary Figure 2). The A > G mutation preferentially targeted a 5′-GGATG-3′ motif in severe cases, the C > T mutation preferentially targeted a 5′-TTCTA-3′ motif in severe cases, and the G > A mutation preferentially targeted a 5′-AAGGG-3′ motif in severe cases.

### Identification of Mutation Hotspots in Different Clinical Outcomes

**FIGURE 2:**
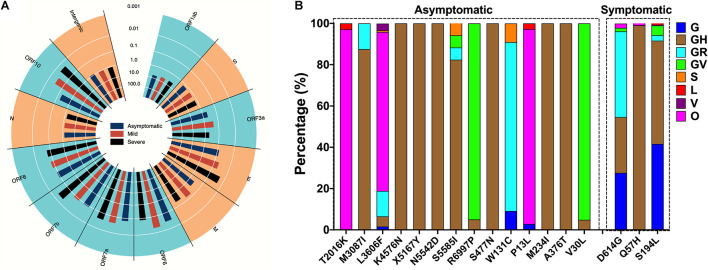
Identification of mutation hotspots in different clinical outcomes. **(A)** Missense mutations of the three clinical outcomes distributed in different genes. **(B)** Major SNV frequency in asymptomatic, mild/moderate, and severe outcomes.

A total of 2,093 mutation sites were found in the three clinical outcomes. According to the results of the chi-square test, 130 missense mutations were significantly different in the three clinical outcomes. These missense mutations were primarily distributed in ORF1ab (56.98%), S (16.28%), ORF3a (11.63%), and N (10.47%) (Figure 2A). The distribution of mutation in the clades was analyzed. In this study, 25 major missense mutation (frequency >5% in at least one of clade) were included in the analysis of distribution in different clades. These mutations were predominantly distributed in GH clade, followed by GR and GV clades (Figure 2B).

To exclude the effect of other variable, including region, gender, and age, multivariate logistic regression was used to analyze the association between mutations and clinical outcomes. The result revealed that 24 missense mutations were correlated with clinical outcome (Table 1). Twenty-one mutations were related to asymptomatic outcomes; these mutations were mainly distributed in ORF1ab and N genes, followed by ORF3a, ORF8, and ORF10 genes. Three mutations were associated with severe outcomes in S, ORF3a, and N genes separately.

**TABLE 1:** SARS-CoV-2 mutations correlated to clinical outcome.

Protein name	Protein mutation	Asymptomatic	Mild/Moderate	Severe/ICU/Decea sed	Chi-square test *p-*value	Adjusted *p-*value^a^
ORF1ab	I300F	2.87% (6/209)	0.00% (6/209)	0.13% (6/209)	6.69E-06	0.01
ORF1ab	A812D	2.87% (6/209)	5.00% (6/209)	2.05% (6/209)	8.45E-06	0.001
ORF1ab	T2016K	9.09% (19/209)	3.24% (19/209)	0.51% (19/209)	4.37E-15	0.000
ORF1ab	M3087I	6.22% (13/209)	0.88% (13/209)	0.00% (13/209)	3.82E-13	0.002
ORF1ab	L3606F	40.19% (84/209)	5.00% (84/209)	0.00% (84/209)	1.15E-78	0.000
ORF1ab	K4576N	6.22% (13/209)	0.29% (13/209)	0.00% (13/209)	8.03E-15	0.003
ORF1ab	I4683T	3.35% (7/209)	0.29% (7/209)	0.00% (7/209)	3.33E-07	0.035
ORF1ab	L5129F	3.35% (7/209)	2.06% (7/209)	0.13% (7/209)	8.63E-06	0.003
ORF1ab	X5167Y	6.22% (13/209)	0.29% (13/209)	0.00% (13/209)	8.03E-15	0.003
ORF1ab	N5542D	6.22% (13/209)	0.29% (13/209)	0.00% (13/209)	8.03E-15	0.003
ORF1ab	S5585I	6.70% (14/209)	0.29% (14/209)	0.26% (14/209)	5.19E-14	0.000
ORF1ab	R6997P	9.09% (19/209)	0.00% (19/209)	0.26% (19/209)	1.68E-21	0.004
S	S477N	6.22% (13/209)	0.29% (13/209)	0.00% (13/209)	8.03E-15	0.003
S	D614G	58.85% (123/209)	91.47% (123/209)	90.90% (123/209)	2.84E-33	0.000
ORF3a	Q57H	10.05% (21/209)	5.29% (21/209)	39.36% (21/209)	8.05E-25	0.000
ORF3a	W131C	4.78% (10/209)	0.00% (10/209)	0.13% (10/209)	6.85E-11	0.013
ORF8	L84S	2.87% (6/209)	2.94% (6/209)	1.15% (6/209)	4.68E-04	0.000
N	P13L	9.57% (20/209)	3.53% (20/209)	0.51% (20/209)	4.79E-16	0.000
N	S194L	1.44% (3/209)	0.29% (3/209)	7.44% (3/209)	1.14E-04	0.005
N	S202N	0.48% (1/209)	2.06% (1/209)	0.13% (1/209)	9.62E-04	0.001
N	M234I	6.22% (13/209)	0.29% (13/209)	0.00% (13/209)	8.03E-15	0.003
N	A376T	6.22% (13/209)	0.29% (13/209)	0.00% (13/209)	8.03E-15	0.003
N	D377Y	4.31% (9/209)	0.00% (9/209)	0.13% (9/209)	1.28E-09	0.018
ORF10	V30L	9.57% (20/209)	0.00% (20/209)	0.13% (20/209)	5.01E-24	0.004

*^*a*^Region, gender, and age were included as variable, multivariate logistic regression was used to analysis the relation between mutations and clinical outcome, the adjusted *p*-value was presented here.*

### Dynamics Distribution of Clinical Outcome-Associated Mutations Over Time

**FIGURE 3:**
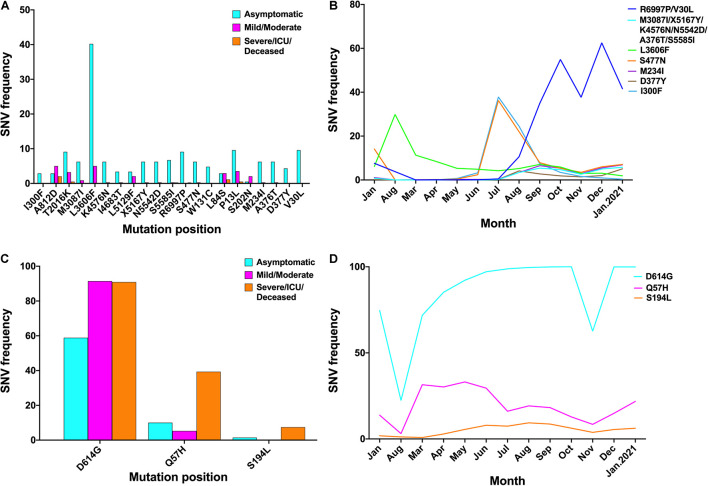
Dynamics distribution of major symptom-associated mutations. **(A)** Mutation sites in asymptomatic cases that were higher than those in symptomatic cases, *p* < 0.05. **(B)** Major SNV frequency of **(A)** over time. **(C)** Mutation sites in symptomatic cases that were higher than those in asymptomatic cases, *p* < 0.05. **(D)** Major SNV frequency of **(C)** over time. SNV, single-nucleotide variant.

Twenty-one major mutations were significantly higher in asymptomatic cases than symptomatic cases (Figure 3A). R6997P and V30L co-occurred and rapidly increased starting in July 2020; M3087I, X5167Y, K4576N, N5542D, A376T, and S5585I co-occurred and gradually increased; and S477N started to rise in June but decreased again in July (Figure 3B). Three major mutations were significantly higher in symptomatic cases than asymptomatic cases (Figure 3C). D614G increased from 74.6% to 99.9%; T265I, Q57H, R203K, and G204R started to increase in March and gradually decreased in July (Figure 3D).

### Single-Nucleotide Variants Associated With RNA-Dependent RNA Polymerase Mutations in Different Clinical Outcomes

**FIGURE 4:**
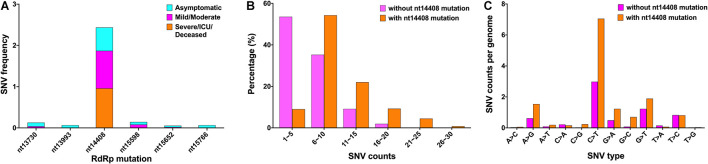
SNVs associated with RdRp mutation in different clinical outcomes. **(A)** Major mutation sites of RdRp in the three clinical outcomes. **(B)** Percentage of genome of different SNV count ranges in the “without nt14408 mutation” and “with nt14408 mutation” groups. **(C)** SNV counts per genome of different SNV types in “without nt14408 mutation” and “with nt14408 mutation” groups. SNV, single-nucleotide variant; RdRp, RNA-dependent RNA polymerase.

Results showed that 142 mutations occurred in the RdRp. Moreover, the incidence rate of nt14408 mutation, which was distributed in asymptomatic cases (56.5%), mild/moderate cases (91.2%), and severe/ICU/deceased cases (95.9%), was the highest (Figure 4A). The 209,551 sequences were divided into “with nt14408 mutation” and “without nt14408 mutation” groups, and the SNV count was calculated and divided into six ranges. According to the results of the chi-square test, 1–5 (*p* < 0.000), 6–10 (*p* < 0.000), 11–15 (*p* < 0.000), 16–20 (*p* < 0.000), and 21–25 (*p* = 0.03) were significantly difference between “with nt14408 mutation” and “without nt14408 mutation” groups; the “without nt14408 mutation” group was primarily distributed in 1–5; and the “with nt14408 mutation” group was mostly distributed in 6–10 and 11–15 (Figure 4B). The SNV type affected by nt14408 mutation was also analyzed. The counts of A > G, C > T, G > A, G > C, and G > T in the “with nt14408 mutation” group were higher than those in the “without nt14408 mutation” group (Figure 4C).

### To Predict the Clinical Outcome Among Severe Acute Respiratory Syndrome Coronavirus 2 and Pandemic Strains

**FIGURE 5:**
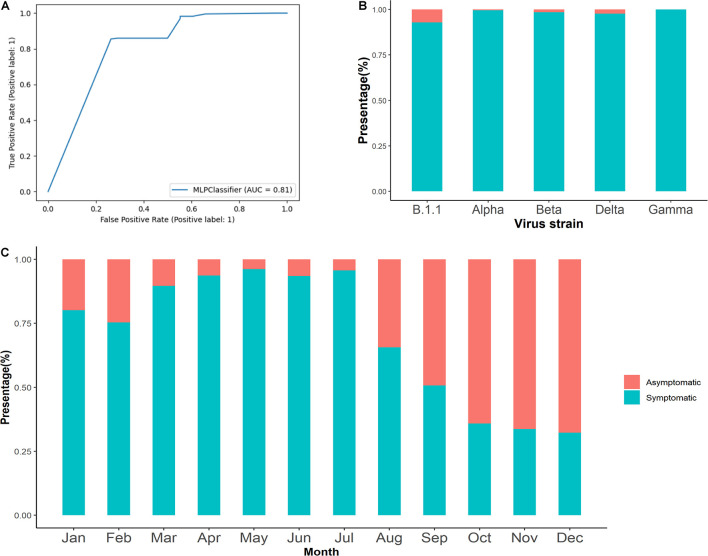
To predict the clinical outcome among SARS-CoV-2 and pandemic strains. **(A)** The ROC curve of the model calculated with the 1,329 samples. **(B)** The percentage of different clinical outcomes among the B.1.1, Alpha, Beta, Delta, and Gamma strains. **(C)** The percentage of different clinical outcomes from January 1, 2020, to December 31, 2020. SARS-CoV-2, severe acute respiratory syndrome coronavirus 2; ROC, receiver operating characteristic.

To verify the accuracy of the model, sequences were collected with clinical information and input in the model to calculate the clinical outcome, and the accuracy of the model was 93.23%, which means that it was suitable for predicting clinical outcome by using the same kind of dataset. The area under the receiver operating characteristic (ROC) curve was 0.81, which proved that this model had a predictive value (Figure 5A). The Alpha, Beta, and Gamma sequences were downloaded from January 1, 2020, to December 31, 2020, and were input into the model to predict the clinical outcome. The percentage of symptomatic outcomes of Alpha, Beta, Delta, and Gamma strains were 99.57, 98.51, 97.75, and 100%, respectively (Figure 5B). For all sequences from January 1, 2020, to December 31, 2020, the percentage of symptomatic outcomes decreased from 80.10% to 32.28%, while the percentage of asymptomatic outcomes increased from 19.90 to 67.72% (Figure 5C).

### RNA Editing in the Transcriptome of Severe Acute Respiratory Syndrome Coronavirus 2

**FIGURE 6:**
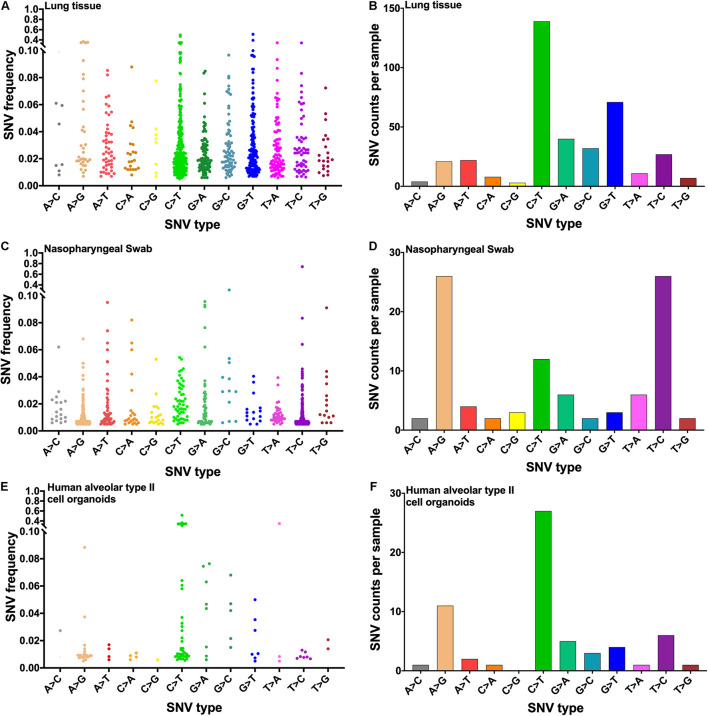
RNA mutation in the transcriptome of SARS-CoV-2. **(A)** Distribution of all SNV types from lung tissues. **(B)** Different SNV counts per sample from lung tissues. **(C)** Distribution of all SNV types from nasopharyngeal swabs. **(D)** Different SNV counts per sample from nasopharyngeal swabs. **(E)** Distribution of all SNV types from human alveolar type II cell organoids. **(F)** Different SNV counts per sample from human alveolar type II cell organoids. SARS-CoV-2, severe acute respiratory syndrome coronavirus 2; SNV, single-nucleotide variant.

The SNV type in the transcriptome of SARS-CoV-2 was analyzed to validate further the RNA editing in the body. Finally, 19 lung tissue samples, seven swab samples, and three human alveolar type II cell organoids samples were qualified for further analysis. In each group, the distribution of all SNV types was different. C > T and G > T mutations were the major SNV types in lung tissues (Figures 6A,B), A > G and T > C SNVs were the main SNV types in nasopharyngeal swabs (Figures 6C,D), and A > G and C > T SNVs were the primary SNV types in human alveolar type II cell organoids (Figures 6E,F). The SNV types in every individual were also analyzed; the SNV types were different (Supplementary Figure 3).

RNA-seq datasets were used to analyze the expression of RNA-editing enzymes based on TPM. The expression levels of ADAR1, APOBEC3C, APOBEC3D, APOBEC3F, APOBEC3G, and APOBEC4 were higher among SARS-CoV-2 infection cases than in those not infected by the virus (Supplementary Figures 4A–C). The expression levels of ADARB1, APOBEC1, and APOBEC3H were higher among severe cases than moderate cases, while the expression levels of ADARB1 and ADARB2 were higher among mild cases than moderate cases (Supplementary Figures 4D–F). Further analysis revealed that the expression of APOBEC3A (*r* = 0.32) was positively correlated with the virus load of SARS-CoV-2 (Supplementary Figure 4G). However, the expression levels of ADARB2 (*r* = −0.29), APOBEC2 (*r* = −0.35), APOBEC4 (*r* = −0.28), and ROMO1 (*r* = −0.34) were negatively correlated with the virus load of SARS-CoV-2 (Supplementary Figures 4H–K).

## Discussion

Thousands of mutations in SARS-CoV-2 have been identified. Some of these mutations have become largely fixed in more recent, geographically defined viral populations (Kaushal et al., 2020; Saha et al., 2020; Sapoval et al., 2020). Thus, these mutations might be the underlying factors of different clinical outcomes, and they may affect the efficacy of antiviral therapies. Mutations in viral genes may have a direct correlation to clinical outcomes (Toyoshima et al., 2020; Nagy et al., 2021; Yildirim et al., 2021). However, the reason that these mutations caused different clinical outcomes remains unknown. In this study, we identified mutation hotspots in the SARS-CoV-2 sequence associated with clinical outcomes, clades, and regions. We found that the mutations correlated with asymptomatic outcomes were mainly in ORF1ab and N genes; especially R6997P and V30L mutations occurred together and were correlated with asymptomatic outcome with high prevalence. The SNV frequency of D614G, S194L, and Q57H, which was found to be highly distributed in the Alpha, Beta, and Gamma, were higher in severe cases than in asymptomatic cases. Furthermore, we found that the frequency of nt14408 mutation, which was found in RdRp gene and was observed to increase the susceptibility to mutations, was higher in severe cases than in asymptomatic cases. Moreover, we established a model that could accurately predict the clinical outcome after SARS-CoV-2 infection.

We identified some mutation hotspots in the SARS-CoV-2 genome that were associated with severe outcomes. For example, the Q57H variant, which was found to occur in the ORF3a protein, had a higher frequency in severe cases (39.36%) than in asymptomatic cases (10.04%). A previous study reported that the Q57H variant could cause dramatic changes in protein structures and decrease the flexibility of domains, thereby enhancing the binding affinities in ORF3a-M and ORF3a-S complexes (Rahman et al., 2021). Other studies demonstrated that the ORF3a protein could induce apoptosis in cells (Ren et al., 2020), similar to SARS-CoV (Law et al., 2005; Waye et al., 2005). However, SARS-CoV-2 ORF3a has a weaker pro-apoptotic activity than SARS-CoV ORF3a. This property of SARS-CoV-2 ORF3a confers certain advantages: SARS-CoV-2 infection can be relatively mild or even asymptomatic outcomes during the early stages, thus allowing the virus to spread more widely. Majumdar and Niyogi (2020) reported that the ORF3a mutation was associated with a higher case fatality rate in SARS-CoV-2 infection. Therefore, further research on these mutations is warranted to determine whether these structural alterations in ORF3a influence protein functions and even virus infectivity.

D614G and S194L were found to be associated with severe outcomes with high frequency worldwide. The D614G variant, which was found to occur in the S protein, has been proved to enhance infection and transmission (Korber et al., 2020; Zhou et al., 2020). Some of the mutation hotspots identified herein, namely, R203K, G204R, L3930F, and V1176F, were also high in severe cases, but significant statistical differences were not found. T265I, which is located in non-structural protein 2 (nsp2), has been observed in 13.83% of all cases worldwide. nsp2 is an important domain that ensures the functional integrity of the mitochondria and responds to cellular stress (Chowdhury et al., 2014). A change from a polar amino acid (threonine) to a non-polar one (isoleucine) can render nsp2 hydrophobic, thereby inducing structural alterations in that domain (Zhang et al., 2020). The R203K and G204R variants co-occurred in the N protein and caused dramatic changes in protein structure [root mean square deviation (RMSD) ≥5.0 Å], thus decreasing the flexibility of the domain (Rahman et al., 2021). More importantly, these mutation hotspots were also found in the three highly transmissible SARS-CoV-2 variants (i.e., Alpha, Beta, and Gamma). These new variants with additional mutation are rapidly spreading in the United Kingdom (Alpha), South Africa (Beta), and Brazil (Gamma). Whether these mutations are associated with clinical outcome needs further study.

Furthermore, we found more mutations associated with asymptomatic outcomes, and mainly in ORF1ab and N genes. For example, the L3606F mutation in non-structural protein 6 (nsp6) was significantly higher in asymptomatic cases (40.19%) than in symptomatic cases (3.39%). nsp6 mutation could affect viral autophagy (Benvenuto et al., 2020), a critical host antiviral defense. The issue of whether L3606F mutation can weaken the autophagy function of nsp6 requires further study. Notably, these major mutations in asymptomatic cases mostly co-occurred and were distributed in different genes. For example, R6997P (ORF1Ab), A222V (S), A220V (N), and V30L (ORF10) co-occurred in 0.5% of all cases in July 2020 and exceeded 50% of all cases by January 2021 worldwide. M3087I (ORF1Ab), K4576N (ORF1Ab), X5167Y (ORF1Ab), N5542D (ORF1Ab), S5585I (ORF1ab), and A376T (N) co-occurred in 0.8% of all cases in January 2020 to 5.7% of known cases in January 2021 worldwide. Thus, the mutation is a cumulative process. This significant result indicated that co-occurring mutations are an important factor for the SARS-CoV-2 pandemic. These mutations may be relevant in designing vaccines.

Depending on these mutation hotspots, we made a model that could predict the clinical outcome. The result has a high accuracy (95.11%), which will be helpful for predicting the epidemic of SARS-CoV-2. With the development of the epidemic, many studies have shown that many COVID-19 patients have no symptoms but can spread the virus to others (Kronbichler et al., 2020; Chen et al., 2021; Ralli et al., 2021). In the early stages of the epidemic, the proportion of symptomatic outcomes was high (Bi et al., 2020) and gradually declined with the epidemic of SARS-CoV-2 according to the predicting result of our model. However, the proportion of symptomatic outcomes among the three highly transmissible SARS-CoV-2 variants (i.e., Alpha, Beta, Delta, and Gamma) was more than 98.50%, which means that these mutant strains had high pathogenicity, and other studies also confirmed the result (Claro et al., 2021; Rahimi and Talebi Bezmin Abadi, 2021). So management of and risk assessment for clinical outcome have also become one of the main difficulties faced by the current epidemic prevention and control measures. Our model will be helpful to predict the proportion of clinical outcome among each strain and take effective measures to control SARS-CoV-2.

Recent studies reported that host-dependent RNA editing (Di Giorgio et al., 2020) and RdRp variant (Pachetti et al., 2020) were associated with SARS-CoV-2 mutation. The process of viral genome mutagenesis includes host-dependent RNA-editing enzymes, RdRp, and spontaneous nucleic acid damages due to physical and chemical mutagens and recombination events. In the study, the frequency of nt14408 mutation, which was in RdRp (also named nsp12), was higher in severe cases (95.90%) than in asymptomatic cases (56.46%). Notably, the “with nt14408 mutation” group had higher mutation counts than the “without nt14408 mutation” group, especially for the C > T, A > G, G > A, and G > T mutations. The RdRp is a multi-domain protein that can catalyze the RNA–template-dependent formation of phosphodiester bonds between ribonucleotides in the presence of divalent metal ions (Bruenn, 2003; Venkataraman et al., 2018). In most viruses, RNA polymerase lacks proofreading capability, with some exceptions such as *Nidovirales* order (to which the *Coronavirus* genus belongs). SARS-CoV-2 shares a higher homology for nsp12 compared with SARS-CoV, suggesting that its function and mechanism of action might be well conserved (Wu et al., 2020). Pachetti et al. (2020) suggested that the RdRp variant was possibly associated with SARS-CoV-2 mutations. In the present work, the frequency of nt14408 mutation in RdRp was higher in severe cases. We speculate that the nt14408 mutation may be one factor of mutations more susceptible for severe cases. The expression levels of ADAR1, APOBEC3C, APOBEC3D, APOBEC3F, APOBEC3G, and APOBEC4 increased after SARS-CoV-2 infection, indicating that ADARs and APOBECs were involved in SARS-CoV-2 infection. We also found that clinical outcomes could affect the expression of ADAR and APOBEC, a process that was probably the main cause of different mutation rates among clinical outcomes.

## Conclusion

These mutation hotspots may be associated with clinical outcome and epidemic. The nt14408 mutation in RdRp and host-dependent RNA editing may be the causes of different mutation frequencies among different clinical cases. The clinical outcome requires attention, which could be used to predict the SARS-CoV-2 epidemic. The model that we made will be helpful for predicting clinical outcome and prevention and control of COVID-19. This study also provides insights into the development of diagnostic and therapeutic strategies for COVID-19.

## Data Availability Statement

The datasets presented in this study can be found in online repositories. The names of the repository/repositories and accession number(s) can be found in the article/Supplementary Material.

## Author Contributions

YLH, JL, and XP conceived the study. PL, BX, and MZ designed the analysis code. XP, LZ, LQ, QW, YW, BX, XX, LL, CY, LW, KH, and YH analyzed the data. YLH and XP wrote the manuscript. All authors have reviewed the manuscript.

## Conflict of Interest

The authors declare that the research was conducted in the absence of any commercial or financial relationships that could be construed as a potential conflict of interest.

## Publisher’s Note

All claims expressed in this article are solely those of the authors and do not necessarily represent those of their affiliated organizations, or those of the publisher, the editors and the reviewers. Any product that may be evaluated in this article, or claim that may be made by its manufacturer, is not guaranteed or endorsed by the publisher.

## Footnotes

1
https://www.gisaid.org/

2
https://www.ncbi.nlm.nih.gov/sra/

## Supplementary Material

Supplementary Figure 1 Analysis of the factors associated with SNV types. **(A)** SNV types distributed in different clades; **(B)** SNV types distributed in different regions. **(C)** SNV types distributed in different ages; **(D)** SNV types distributed in different genders. ^a^*p* < 0.001 represents there is significant difference among the eight clades. ^b^*p* < 0.001 represents there is significant difference among the six regions.

Supplementary Figure 2 Motif of different SNV types in SARS-CoV-2 among different clinical outcomes.

Supplementary Figure 3 Distribution of all SNV types among different samples.

Supplementary Figure 4 Analysis of expression of RNA-editing enzyme in the transcriptome. **(A–C)** Expression of RNA-editing enzyme after SARS-CoV-2 infection and other infection from nasopharyngeal swabs. **(D–F)** Expression of RNA-editing enzyme in the three clinical outcomes from nasopharyngeal swabs. **(G–L)** Correlation of expression levels of ADARB2, APOBEC2, APOBEC3A, APOBEC4, ROMO1, and NRROS with SARS-CoV-2 virus load. ^∗^*p* < 0.05, ^∗∗^*p* < 0.01, ^∗∗∗^*p* < 0.001.
