# Supplementary figures and images for: Emerging Severe Acute Respiratory Syndrome Coronavirus 2 Mutation Hotspots Associated With Clinical Outcomes and Transmission
Source: Front Microbiol. 2021 Oct 18;12:753823. doi: 10.3389/fmicb.2021.753823 (PMC8558435; doi:10.3389/fmicb.2021.753823)

Figure S1

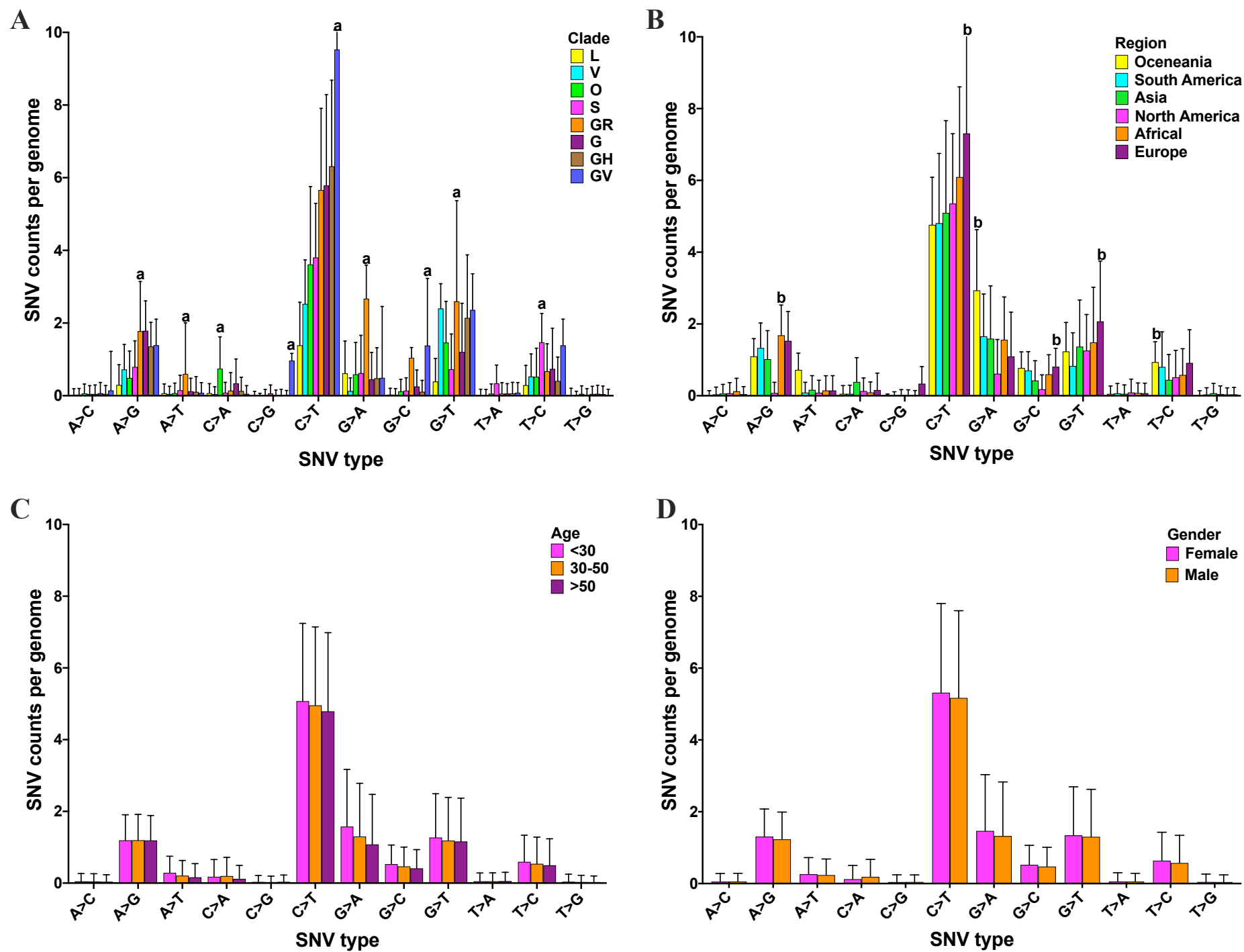

Supplement: Supplementary Figure 1 — Analysis of the factors associated with SNV types. (A) SNV types distributed in different clades; (B) SNV types distributed in different regions. (C) SNV types distributed in different ages; (D) SNV types distributed in different genders. ap < 0.001 represents there is significant difference among the eight clades. bp < 0.001 represents there is significant difference among the six regions. [file Data_Sheet_1.PDF]

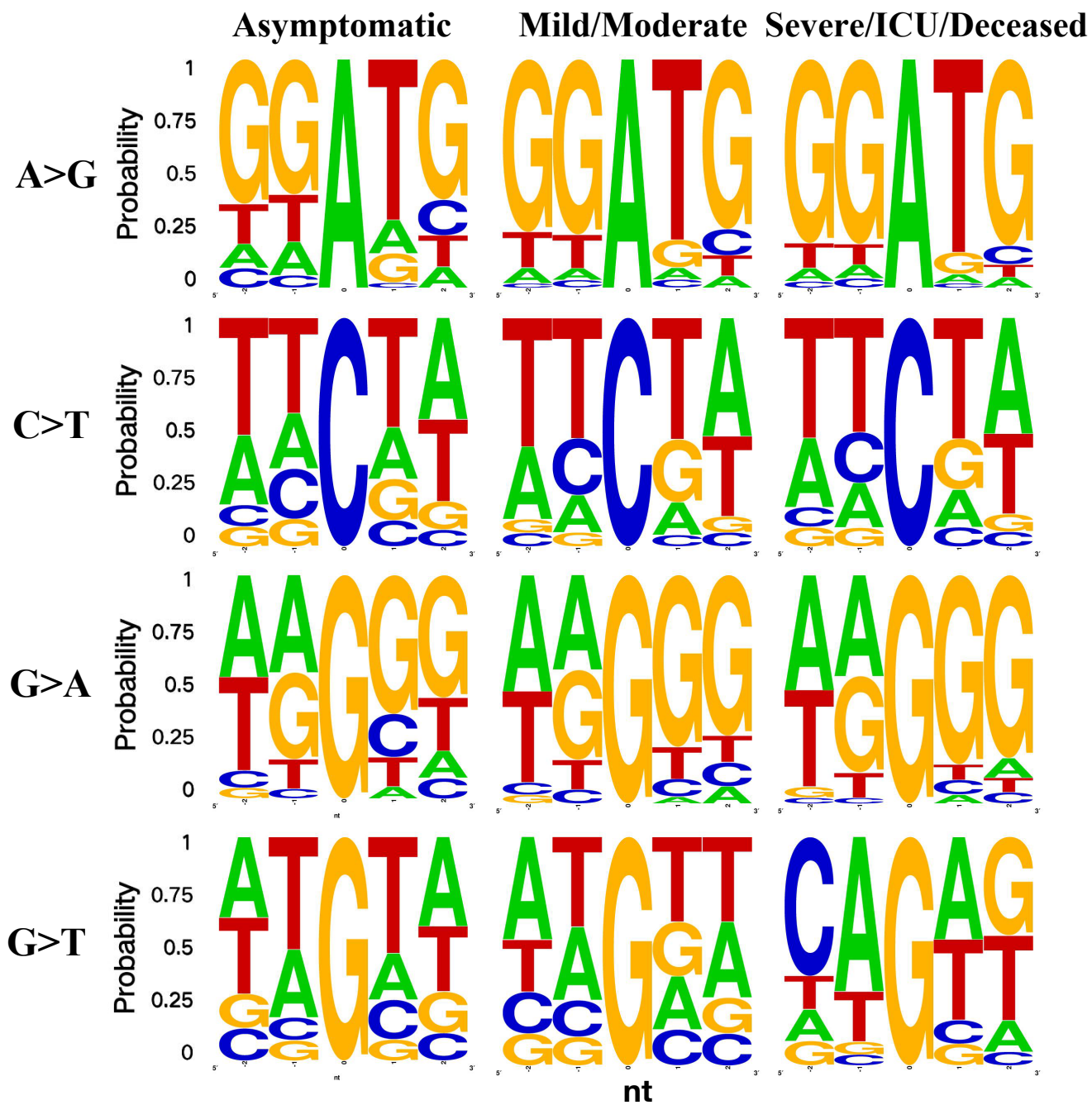

Supplement: Supplementary Figure 2 — Motif of different SNV types in SARS-CoV-2 among different clinical outcomes. [file Data_Sheet_2.PDF]

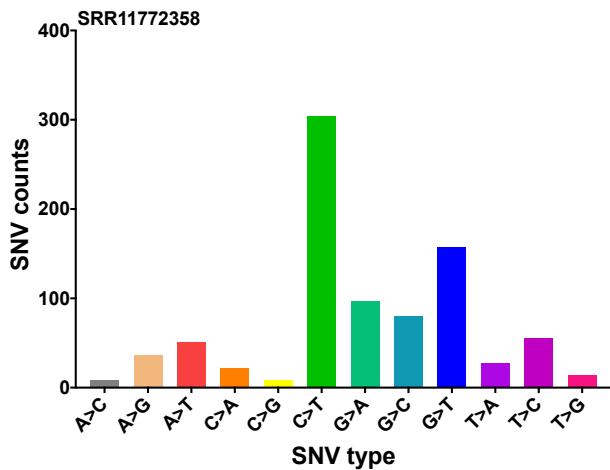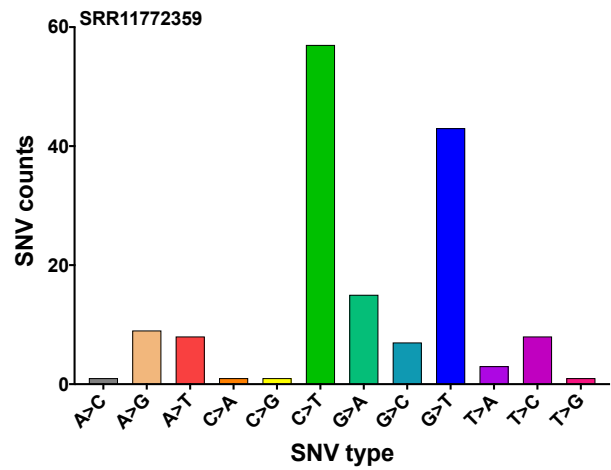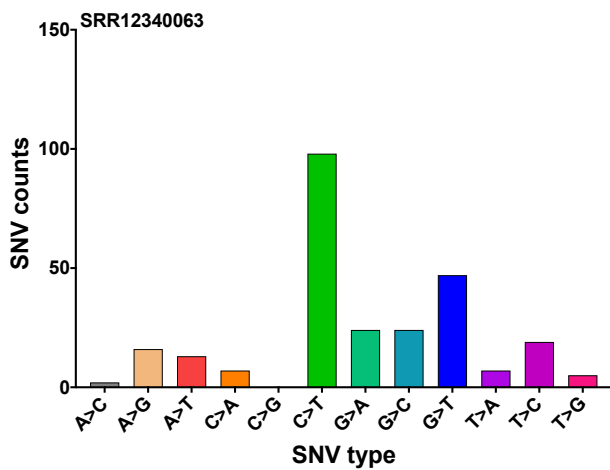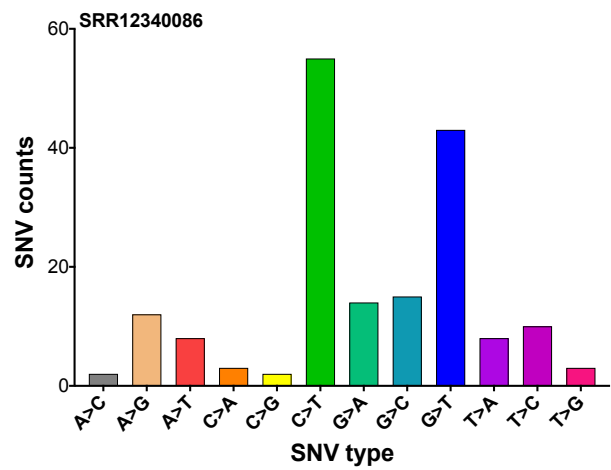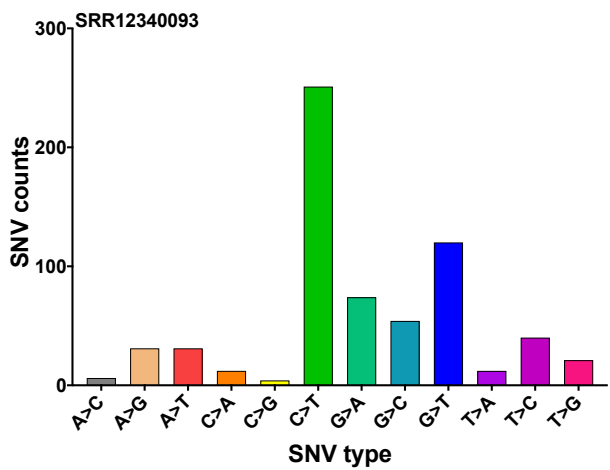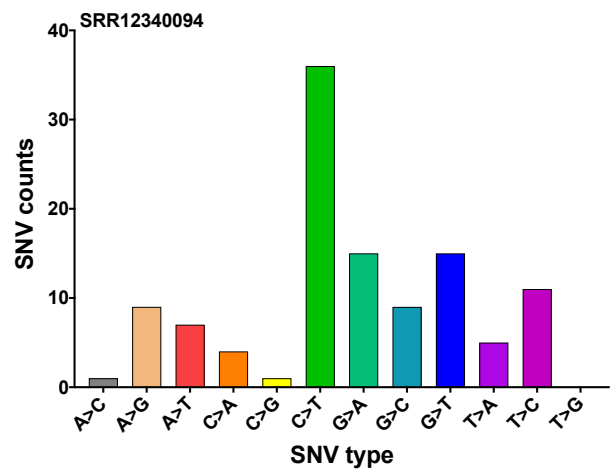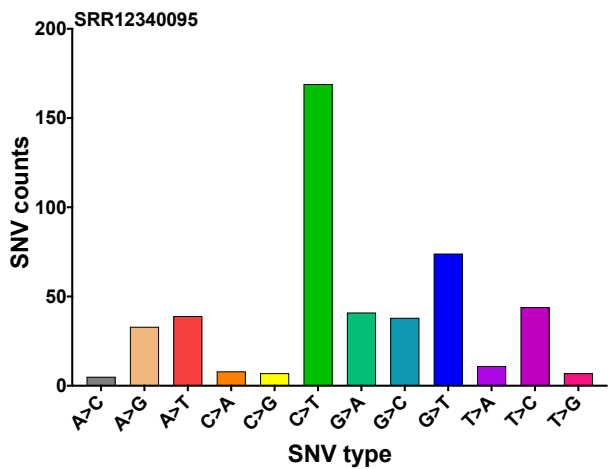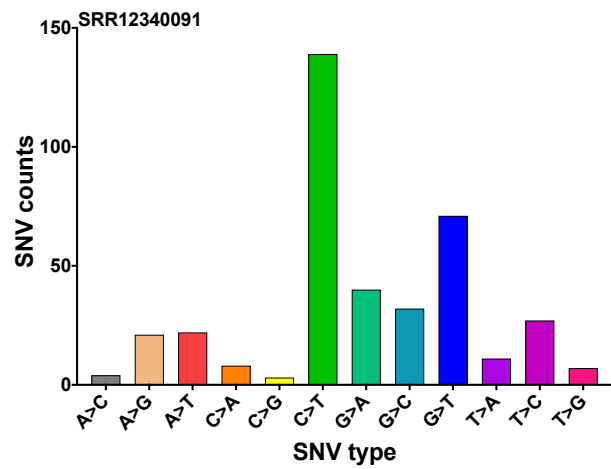

Supplement: Supplementary Figure 3 — Distribution of all SNV types among different samples. [file Data_Sheet_3.PDF]

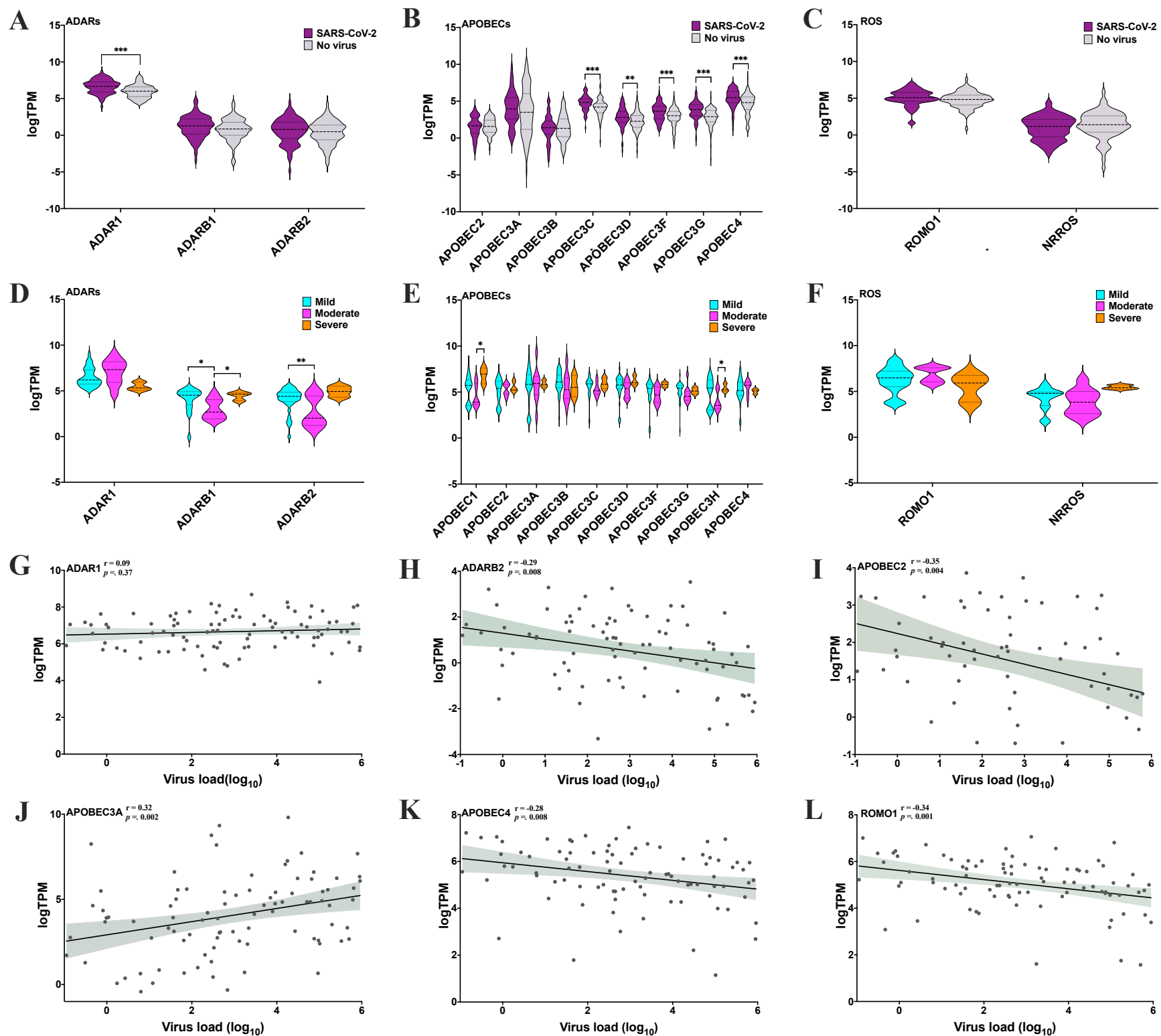

Supplement: Supplementary Figure 4 — Analysis of expression of RNA-editing enzyme in the transcriptome. (A–C) Expression of RNA-editing enzyme after SARS-CoV-2 infection and other infection from nasopharyngeal swabs. (D–F) Expression of RNA-editing enzyme in the three clinical outcomes from nasopharyngeal swabs. (G–L) Correlation of expression levels of ADARB2, APOBEC2, APOBEC3A, APOBEC4, ROMO1, and NRROS with SARS-CoV-2 virus load. ∗p < 0.05, ∗∗p < 0.01, ∗∗∗p < 0.001. [file Data_Sheet_4.PDF]
